# The design and analysis of a new slipper-type hydraulic support

**DOI:** 10.1371/journal.pone.0202431

**Published:** 2018-08-17

**Authors:** Yang Yang, Qingliang Zeng, Jiehan Zhou, Lirong Wan, Kuidong Gao

**Affiliations:** 1 Department of Mechanical and Electrical Engineering, Shandong University of Science and Technology, Qingdao, Shandong, China; 2 Faculty of Information Technology and Electrical Engineering, University of Oulu, Oulu, Finland; Stellenbosch University, SOUTH AFRICA

## Abstract

To improve the safety and the stability of the support under mines and reduce the cost, we design a new slipper-type hydraulic support with energy-efficiency and high reliability. To study its dynamics, we build a reverse kinematics model. We analyze the motion and the force for each component of the new support with a simulation in Matlab/Simulink. The results show that it has appropriate structures with the required four-bar linkages. To compare the performance between the new slipper-type support and the traditional support, we design their mechanics models, deduce their mechanics relations and obtain the force curves for each component of both supports under the same loads. The results prove that the new slipper-type support has less demand on oil pressure for the hydraulic cylinder when working at middle and high positions and it has a larger supporting force and a higher supporting stability which would be more energy-efficient.

## Introduction

Hydraulic supports [1–10] are important equipment in mechanized coal mining systems [11]. It mainly supports the working face in the mine, cooperating with the shearer and scraper. The stability and reliability of a hydraulic support plays an important role to not only guarantee the safe operation of the coal mine, but also to improve the mine’s efficiency and economic performance.

Because of its importance, hydraulic support attracts much attention from both researchers and practitioners of mechanized coal mining. Since the first support was developed in England in 1854, many studies [12–22] have been done on the design, manufacturing, and utilization of hydraulic supports. Oblak et al. [23] proposed a procedure to optimize two groups of parameters of a hydraulic support based on mathematical programming methods. Alehossein et al. [24] performed the stress analysis of longwall top coal caving. Khanal et al. [25] performed the analysis to the mine of Longwall Top Coal Caving by a continuum mechanics finite element solver. González-Nicieza et al. [26] presented a support system for determining the maximum pressure in anthracite mining. Hua et al. [27] presented an effective control method by analyzing the major factors influencing the support stability in a case study of a four-pole hydraulic support. Tu et al. [28] presented a method for controlling the support stability in the mining of steep coal seams by analyzing the instable mode of sliding and tripping of a support. Zhao et al. [29] provided a theoretical reference for the fatigue design of welded structures of a hydraulic support through finite element analysis and fatigue tests. Witek et al. [30] presented a shield on a hydraulic cushion and simulated the ground with different load-bearing capacities, and concluded that the type of base support influences the stress distribution in the shield. Yu et al. [31] studied the dynamic bearing and adaptability of a hydraulic support in a coal caving with a great mining height. Wang et al. [32] analyzed the stress on doubly-telescopic props of a hydraulic support based on the mixed structured and unstructured finite element mesh and analyzed the stability of props through buckling analysis. The above mentioned works provide the new technical standards for designing hydraulic cylinders and present the theories and methods for improving traditional support design. But those studies are based on enhancing existing supports rather than designing a new support.

The actual working face in the mine is complex and has different types of supports for meeting different conditions. The supports which have been in use could be divided into the following categories. In terms of types, support could be categorized into supporting-type hydraulic support, shield-type hydraulic support, and standing shield hydraulic support. In terms of support weight, support could be categorized into light, medium, and heavy hydraulic support. In terms of support height, support could be categorized into thin seam, medium seam, and thick seam support. The medium seam support can be further subdivided into overall height mining hydraulic support, slicing mining hydraulic support, and top coal caving hydraulic support. The highest working height in the world can reach 8.8 meters. In terms of usage, support could be categorized into termination point hydraulic support and intermediate hydraulic support. Currently, hydraulic support has been well developed and more attention is being paid to support structure optimization, functional upgrading, energy saving, and increasing output. Hou et al. [33] designed a highly reliable and low-energy consumption hydraulic system with the use of large-flow cartridge relief valves. Song et al. [34] designed a semi-thin coal unloading alternating push-powered support and succeeded at the unattended operation in a thin seam inclined short wall face. Yu et al. [35] carried out kinematics simulation for 6-SPS hydraulic support and verified the rationality of parallel mechanism of hydraulic supports. Zhang et al. [36] proposed a hydraulic support with reverse four connecting rod mechanism with better performance based on motion simulation analysis. Based on the parallel topological structure, Wang et al. [37–39] designed three-DOF double parallel hydraulic support with yield and pressure relief while preventing support from collapsing: their support can bear the forces from three directions and has good stability and adaptability in large dip angle. These creative design concepts freshen up the development of hydraulic support. But affected by the economic situation at home and abroad, the coal industry has been in recession. Improving mining rate and reducing energy consumption and mining costs while improving the safety and reliability of supporting is still the focus of coal companies under the new situation. Based on the summary and reference of previous design and research on parallel mechanism and hydraulic support, this paper presents a design of slipper-type support with a new type adjusting system.

Hydraulic support is physical equipment used in coal mining, but it can be understood and analyzed as a set of parallel mechanisms which takes the bar system as the main body. Prebil et al. [40] optimized the four-bar mechanism of hydraulic support through global optimal solution to find the optimal values of the mechanism link lengths. Dong et al. [41] modelled a new type of top-coal caving hydraulic support as a plane mechanism to study the structure and kinetic characteristic of support by solving its equations of motion. The environment characteristic and load-bearing characteristic of equipment can be explored more accurately by analyzing from the perspectives of design and mathematical model. To obtain the precise motion and load-bearing characteristics of the new slipper-type hydraulic support, this paper presents a reverse kinematics model and load-bearing mechanical models of the support based on the theory of mechanisms, and analyzes the support through building and solving a numerical simulation model.

The contributions of the paper include:

- A new slipper-type hydraulic support design that improves the safety and the stability of the support while reducing cost.
- To study the dynamics of the new type hydraulic support, we created its reverse kinematics mathematical model. The numerical solution model was built in Matlab/simulink in order to get accurate data, through which the exact motion state of each component of the hydraulic support can be obtained when the driving parameters are set.
- We have proved that the new slipper-type support has smaller demand on oil pressure and larger supporting force. The new support would be more energy-saving than the traditional one.

The remainder of the paper is organized as follows: Section 2 introduces the structure of the new slipper-type hydraulic support, and its motion analysis. After obtaining the kinematic characteristics, Section 3 compares the mechanics models of the new slipper-type support and the traditional support. Section 4 compares the stress condition of the two kinds of hydraulic supports, and analyzes the causes of differences between them. Section 5 shows some related work and our conclusion.

## Design scheme and mechanism analysis

### Design scheme

To ensure reasonable stress and reliable operation, the distance between the front endpoint of the top beam and the coal wall should be minimized. The new slipper-type support described in this paper uses the four-bar linkage of traditional support, but its new design has many advantages over the traditional supports.

**Fig 1:**
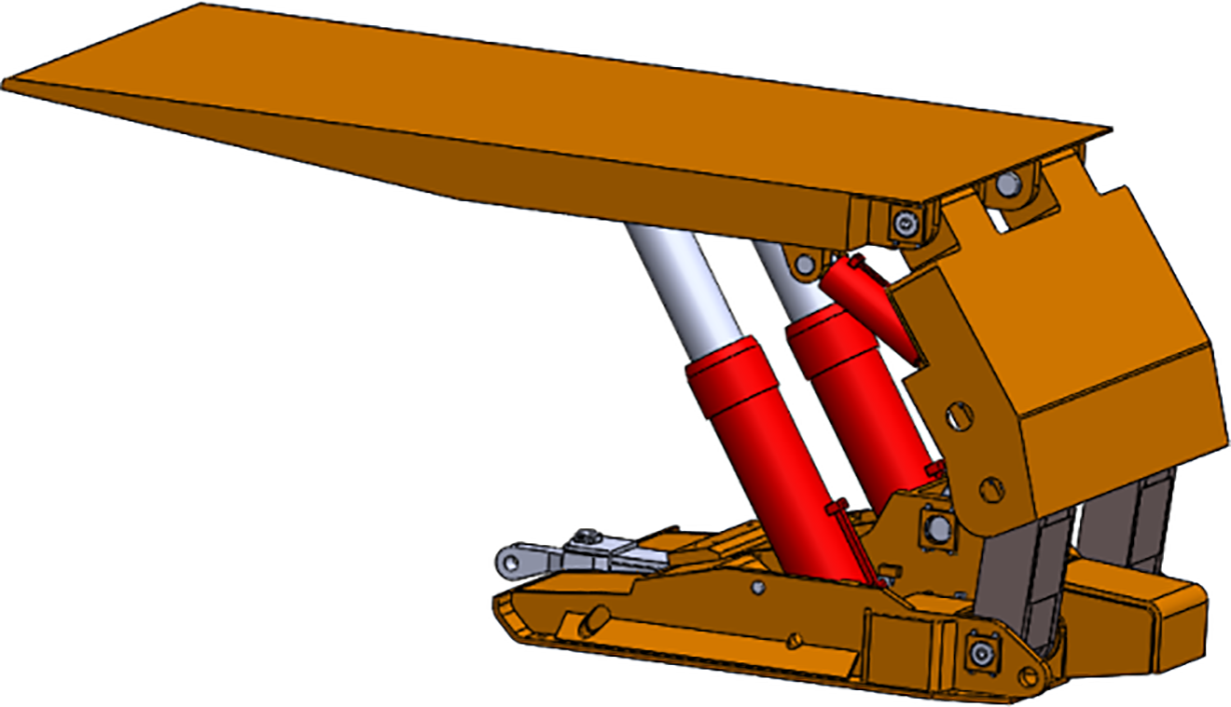
Structure of traditional support.

**Fig 2:**
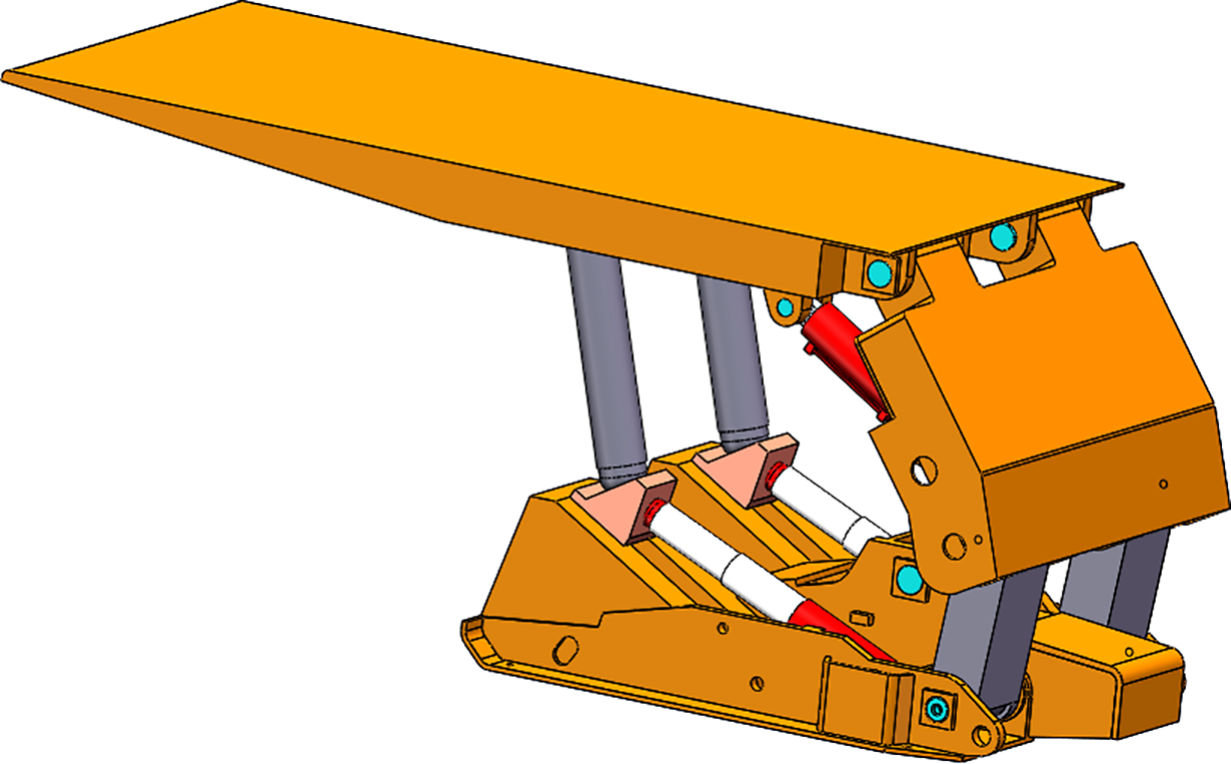
Structure of slipper-type support.

Based on the classical structure of the traditional support, we improved the approach of adjusting the support. The supporting structure of the main body was changed from the traditional prop shown in Fig 1 to an adjusting structure composed of vertical bar, slider, adjusting hydraulic cylinder, and the inclined ramp. The overall structure of the slipper-type hydraulic support is shown in Fig 2.

In the new design we removed the lower needling and added the inclined ramp for sliders. The vertical bar is connected with the adjusting cylinder by setting a slider with a bidirectional needling mechanism. The extension of the adjusting cylinder drives the slither of the slider along the inclined ramp on the base. The slither of the slider drives the pillar swinging around the upper pillar. The height of the support will be adjusted based on the difference of the height between the lowest and highest height of the swinging vertical bar. Compared with the traditional prop support, the advantage of the slipper-type support composed of vertical bar, slider, adjusting hydro-cylinder and inclined ramp is that during raising and roof supporting, the slipper-type support would effectively save electricity for pump stations, including the consumption of the high-pressure emulsion.

### Mechanism principle

The slipper-type support belongs to the two-pillar shield-type support in structure. It consists of top beam, vertical bar, slider, balance jack, shield beam, adjusting hydro-cylinder, front linkage, rear linkage and base. The whole support has four motion transmission rings, of which the adjusting cylinder (the driving part) and the balance jack belong to different rings. Therefore the slipper-type support could be regarded as a set of parallel mechanisms. In terms of the characteristics of its work and structural motion, the slipper-type support could be simplified to a set of planar parallel mechanism.

#### DOF analysis of slipper-type support

According to the theory of parallel mechanism and the redundant constraints formula of the DOF (Grübler-Kutzbach formula)

$$DOF=s\times (n-m-1)+\sum_{i=1}^{m}f_{i}-\nu -q$$ (1)

Where *s* is the order of mechanism, *s* = 6-*λ* if *λ* is the number of general constraints, *n* is the component number in the mechanism, *m* is the number of kinematic-pair of the mechanism, *f*_*i*_ is the number of relative degrees of freedom of *i*^*th*^ kinematic pair, *υ* is the number of virtual constraints, *q* is the local degree of freedom of the mechanism.

**Fig 3:**
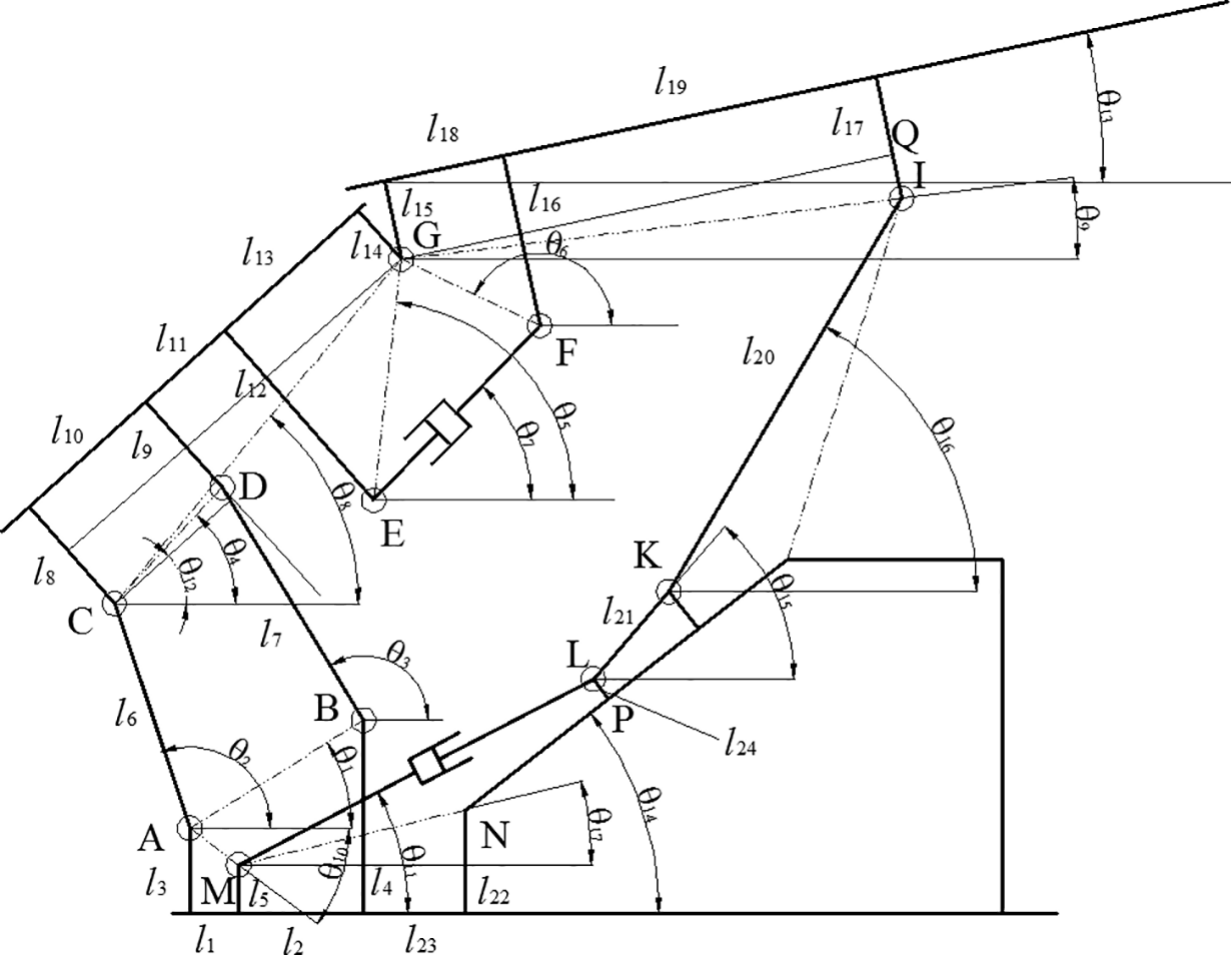
The schematic diagram of the slipper-type support.

Based on the schematic diagram of the slipper-type support shown in Fig 3, the mechanism component number *n* = 9, kinematic pair number *m* = 14, general constraint number *λ* = 3, so the order of mechanism s = 6-*λ* = 3, the virtual constraint *υ* = 0, the local degree of freedom *q* = 0.

Taking the above parameters into formula (1), the DOF of slipper-type hydraulic support can be obtained:

DOF=3×(9‑14‑1+14)=2 (2)

The slipper-type hydraulic support has two driving parts: the adjusting hydro-cylinder and the balance jack. The number of driving parts equals the number of DOF of the mechanism. For instance, the instantaneous pose could be calculated if the stroke of driving parts is given, or the position of the driving parts could be calculated by its poses. Thus, we could calculate and so control and adjust the slipper-type hydraulic support according to the actual situation of the working face.

**Building the inverse solution mathematical model of the slipper-type support.** The bars shown in Fig 3 could be equivalent to bar vectors, and the vector sum of the closed vector polygon is 0. We obtain a set of vector equations (i.e., the motion position model of the support) using the four vector rings of A-B-D-C, E-F-G, A-C-G-Q-I-K-L-M, and M-L-P-N, that is:

$$\left\{ \begin{array}{l} \vec{AB}+\vec{BD}=\vec{AC}+\vec{CD}\\ \vec{EG}+\vec{GF}=\vec{EF}\\ \vec{AC}+\vec{CG}+\vec{GQ}+\vec{QI}+\vec{IK}+\vec{KL}=\vec{AM}+\vec{ML}\\ \vec{ML}+\vec{LP}=\vec{MN}+\vec{NP} \end{array}\right.$$ (3)

In solving the reverse solution, giving the height of support H and dip angle of top beam *θ*_13_, then we could calculate the length of the slider jack and equilibrium jack (*s*_1_, *s*_2_). Now *H* and θ_13_ are given, we need to solve <*θ*_2_, *θ*_3_,*θ*_7_,*θ*_11_,*θ*_12_,*θ*_16_, *s*_2_,*s*_3_>. If the top beam is parallel with the base (i.e., *θ*_13_ = 0), the top face of top beam is horizontal, H becomes the height of the top face of top beam, i.e., sin*θ*_13_ = 0, cos*θ*_13_ = 1.

⇒ *H* = *s*_1_·sin*θ*_11_+*l*_KL_·sin*φ*_4_+ *l*_IK_·sin*θ*_16_+ *l*_17_+ *l*_5_

⇒ *s*_1_ = (*H* -*l*_KL_·sin*φ*_4_- *l*_IK_·sin*θ*_16_- *l*_17_- *l*_5_)/sin*θ*_11_

Where $\varphi_{1}=\arctan\frac{l_{4}-l_{3}}{l_{1}+l_{2}},$ $\varphi_{2}=\arctan\frac{l_{3}-l_{5}}{l_{1}},$ *φ*_3_ = *θ*_14_, *φ*_4_ = *θ*_15_, $\varphi_{5}=\arctan\frac{l_{8}-l_{9}}{l_{10}},$ $\varphi_{6}=\arctan\frac{l_{12}-l_{14}}{l_{13}},$ $\varphi_{7}=\arctan\frac{l_{8}-l_{14}}{l_{10}+l_{11}+l_{13}},$ $\varphi_{8}=\arctan\frac{l_{17}-l_{15}}{l_{18}+l_{19}},$ $\varphi_{9}=\frac{\pi }{2}+\arctan\frac{l_{18}}{l_{16}-l_{15}},$ $\varphi_{10}=\arctan\frac{l_{22}-l_{5}}{l_{2}+l_{23}}.$ The final position reverse model is obtained by further simplification:

$$\left\{ \begin{array}{l} l_{AB}\cdot \cos\varphi_{1}+l_{BD}\cdot \cos\theta_{3}=l_{AC}\cdot \cos\theta_{2}+l_{CD}\cdot \cos(\theta_{12}+\varphi_{5})\\ l_{AB}\cdot \sin\varphi_{1}+l_{BD}\cdot \sin\theta_{3}=l_{AC}\cdot \sin\theta_{2}+l_{CD}\cdot \sin(\theta_{12}+\varphi_{5})\\ l_{EG}\cdot \cos(\theta_{12}+\varphi_{6})-l_{GF}\cdot \cos(\varphi_{9}+\pi /2)=s_{2}\cdot \cos\theta_{7}\\ l_{EG}\cdot \sin(\theta_{12}+\varphi_{6})-l_{GF}\cdot \sin(\varphi_{9}+\pi /2)=s_{2}\cdot \sin\theta_{7}\\ l_{AC}\cdot \cos\theta_{2}+l_{CG}\cdot \cos(\theta_{12}+\varphi_{7})+l_{GQ}-l_{KL}\cdot \cos\varphi_{4}=l_{AM}\cdot \cos\varphi_{2}+s_{1}\cdot \cos\theta_{11}\\ l_{AC}\cdot \sin\theta_{2}+l_{CG}\cdot \sin(\theta_{12}+\varphi_{7})-l_{QI}=-l_{AM}\cdot \sin\varphi_{2}+H-l_{17}-l_{5}\\ s_{1}\cdot \sin\theta_{11}/\cot\theta_{11}+l_{LP}\cdot \sin\varphi_{3}=l_{MN}\cdot \cos\varphi_{10}+s_{3}\cdot \cos\varphi_{3}\\ s_{1}\cdot \sin\theta_{11}-l_{LP}\cdot \cos\varphi_{3}=l_{MN}\cdot \sin\varphi_{10}+s_{3}\cdot \sin\varphi_{3} \end{array}\right.$$ (4)

Taking the derivative of time t, the final velocity model is as follows:

$$\left\{ \begin{array}{l} l_{AC}\cdot \sin\theta_{2}\cdot \omega_{2}-l_{BD}\cdot \sin\theta_{3}\cdot \omega_{3}+l_{CD}\cdot \sin(\theta_{12}+\varphi_{5})\cdot \omega_{12}=0\\ l_{AC}\cdot \cos\theta_{2}\cdot \omega_{2}-l_{BD}\cdot \cos\theta_{3}\cdot \omega_{3}+l_{CD}\cdot \cos(\theta_{12}+\varphi_{5})\cdot \omega_{12}=0\\ s_{2}\cdot \sin\theta_{7}\cdot \omega_{7}-l_{EG}\cdot \sin(\theta_{12}+\varphi_{6})\cdot \omega_{12}-v_{2}\cdot \cos\theta_{7}=0\\ -s_{2}\cdot \cos\theta_{7}\cdot \omega_{7}+l_{EG}\cdot \cos(\theta_{12}+\varphi_{6})\cdot \omega_{12}-v_{2}\cdot \sin\theta_{7}=0\\ -l_{AC}\cdot \sin\theta_{2}\cdot \omega_{2}+s_{1}\cdot \sin\theta_{11}\cdot \csc\theta_{11}{}^{2}\cdot \omega_{11}-l_{CG}\cdot \sin(\theta_{12}+\varphi_{7})\cdot \omega_{12}+l_{IK}\cdot \sin\theta_{16}\cdot \omega_{16}\\ +l_{IK}\cdot \cos\theta_{16}\cdot \cot\theta_{11}\cdot \omega_{16}=v_{H}\cdot \cot\theta_{11}\\ l_{AC}\cdot \cos\theta_{2}\cdot \omega_{2}+l_{CG}\cdot \cos(\theta_{12}+\varphi_{7})\cdot \omega_{12}=v_{H}\\ s_{1}\cdot \sin\theta_{11}\cdot \csc\theta_{11}{}^{2}\cdot \omega_{11}+l_{IK}\cdot \cos\theta_{16}\cdot \cot\theta_{11}\cdot \omega_{16}+v_{3}\cdot \cos\varphi_{3}=v_{H}\cdot \cot\theta_{11}\\ l_{IK}\cdot \cos\theta_{16}\cdot \omega_{16}+v_{3}\cdot \sin\varphi_{3}=v_{H} \end{array}\right.$$ (5)

**Fig 4:**
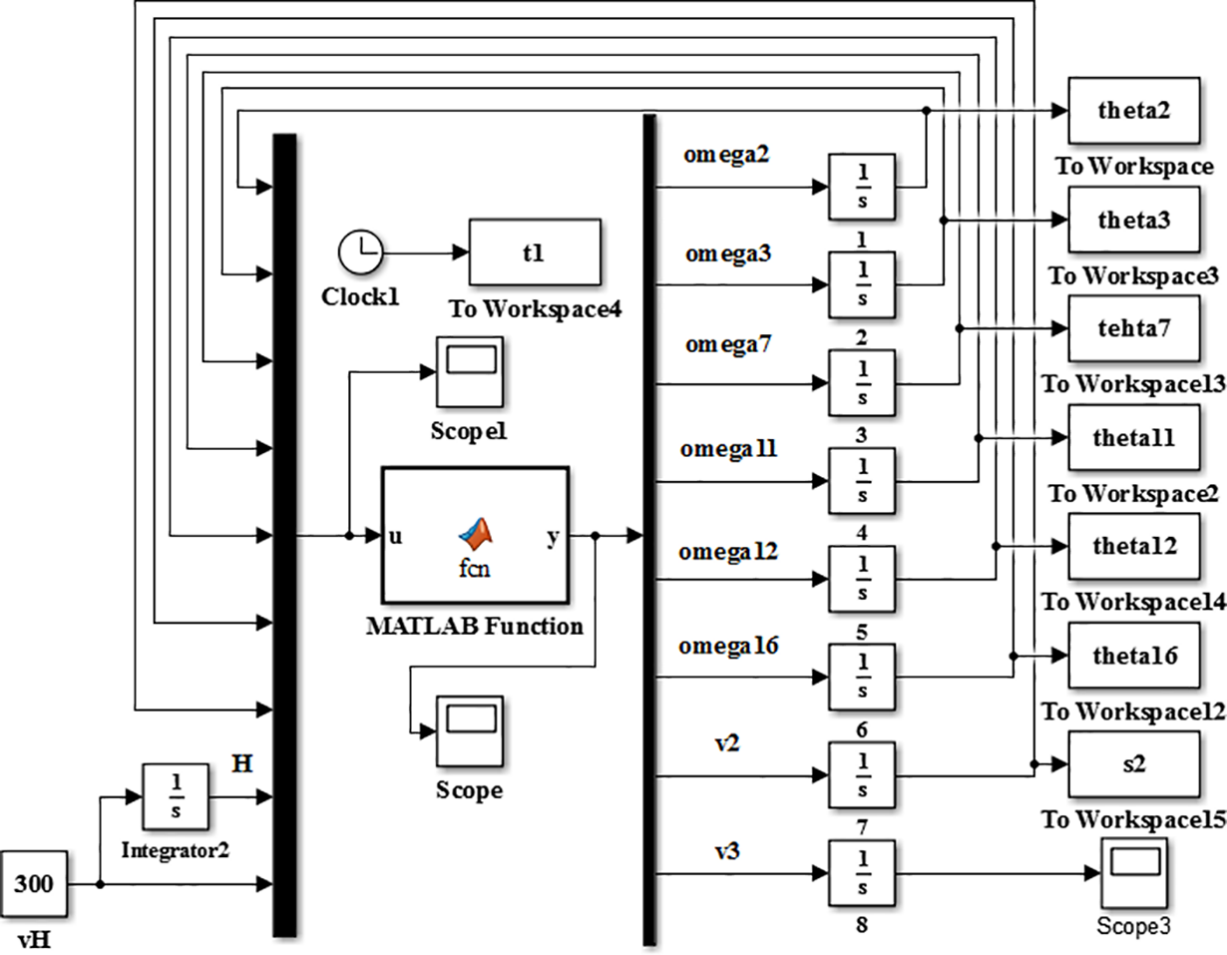
The speed simulation model of the slipper-type support.

The speed simulation model of the slipper-type support is shown in Fig 4 in Simulink. Take the derivative of time t of the velocity model of support reverse model, the acceleration model of support can be obtained based on the variables of (*ε*_2_, *ε*_3_, *ε*_7_, *ε*_11_, *ε*_12_, *ε*_16_, *a*_2_, *a*_3_):

$$\left\{ \begin{array}{l} l_{AC}\cdot \sin\theta_{2}\cdot \varepsilon_{2}-l_{BD}\cdot \sin\theta_{3}\cdot \varepsilon_{3}+l_{CD}\cdot \sin(\theta_{12}+\varphi_{5})\cdot \varepsilon_{12}=-l_{AC}\cdot \cos\theta_{2}\cdot \omega_{2}{}^{2}+l_{BD}\cdot \cos\theta_{3}\cdot \omega_{3}{}^{2}-l_{CD}\cdot \sin(\theta_{12}+\varphi_{5})\cdot \omega_{12}{}^{2}\\ l_{AC}\cdot \cos\theta_{2}\cdot \varepsilon_{2}-l_{BD}\cdot \cos\theta_{3}\cdot \varepsilon_{3}+l_{CD}\cdot \cos(\theta_{12}+\varphi_{5})\cdot \varepsilon_{12}=l_{AC}\cdot \sin\theta_{2}\cdot \omega_{2}{}^{2}-l_{BD}\cdot \sin\theta_{3}\cdot \omega_{3}{}^{2}+l_{CD}\cdot \sin(\theta_{12}+\varphi_{5})\cdot \omega_{12}{}^{2}\\ s_{2}\cdot \sin\theta_{7}\cdot \varepsilon_{7}-l_{EG}\cdot \sin(\theta_{12}+\varphi_{6})\cdot \varepsilon_{12}-a_{2}\cdot \cos\theta_{7}=-2v_{2}\cdot \sin\theta_{7}\cdot \omega_{7}-s_{2}\cdot \cos\theta_{7}\cdot \omega_{7}{}^{2}+l_{EG}\cdot \cos(\theta_{12}+\varphi_{6})\cdot \omega_{12}{}^{2}\\ -s_{2}\cdot \cos\theta_{7}\cdot \varepsilon_{7}+l_{EG}\cdot \cos(\theta_{12}+\varphi_{6})\cdot \varepsilon_{12}-a_{2}\cdot \sin\theta_{7}=2v_{2}\cdot \cos\theta_{7}\cdot \omega_{7}-s_{2}\cdot \sin\theta_{7}\cdot \omega_{7}{}^{2}+l_{EG}\cdot \sin(\theta_{12}+\varphi_{6})\cdot \omega_{12}{}^{2}\\ -l_{AC}\cdot \sin\theta_{2}\cdot \varepsilon_{2}+s_{1}\cdot \sin\theta_{11}\cdot \csc\theta_{11}{}^{2}\cdot \varepsilon_{11}-l_{CG}\cdot \sin(\theta_{12}+\varphi_{7})\cdot \varepsilon_{12}+l_{IK}\cdot \sin\theta_{16}\cdot \varepsilon_{16}+l_{IK}\cdot \cos\theta_{16}\cdot \cot\theta_{11}\cdot \varepsilon_{16}=a_{H}\cdot \cot\theta_{11}\\ -(2v_{H}-l_{IK}\cdot \cos\theta_{16}\cdot \omega_{16})\cdot \csc\theta_{11}{}^{2}\cdot \omega_{11}+l_{AC}\cdot \cos\theta_{2}\cdot \omega_{2}{}^{2}+2s_{1}\cdot \sin\theta_{11}\cdot \csc\theta_{11}{}^{2}\cdot \cot\theta_{11}\cdot \omega_{11}{}^{2}+l_{CG}\cdot \cos(\theta_{12}+\varphi_{7})\cdot \omega_{12}{}^{2}\\ -l_{IK}\cdot \cos\theta_{16}\cdot \omega_{16}{}^{2}+l_{IK}\cdot \sin\theta_{16}\cdot \cot\theta_{11}\cdot \omega_{16}{}^{2}+l_{IK}\cdot \cos\theta_{16}\cdot \csc\theta_{11}{}^{2}\cdot \omega_{11}\cdot \omega_{16}\\ l_{AC}\cdot \cos\theta_{2}\cdot \varepsilon_{2}+l_{CG}\cdot \cos(\theta_{12}+\varphi_{7})\cdot \varepsilon_{12}=a_{H}+l_{AC}\cdot \sin\theta_{2}\cdot \omega_{2}{}^{2}+l_{CG}\cdot \sin(\theta_{12}+\varphi_{7})\cdot \omega_{12}{}^{2}\\ s_{1}\cdot \sin\theta_{11}\cdot \csc\theta_{11}{}^{2}\cdot \varepsilon_{11}+l_{IK}\cdot \cos\theta_{16}\cdot \cot\theta_{11}\cdot \varepsilon_{16}+a_{3}\cdot \cos\varphi_{3}=a_{H}\cdot \cot\theta_{11}-(2v_{H}-l_{IK}\cdot \cos\theta_{16}\cdot \omega_{16})\cdot \csc\theta_{11}{}^{2}\cdot \omega_{11}\\ +2s_{1}\cdot \sin\theta_{11}\cdot \csc\theta_{11}{}^{2}\cdot \cot\theta_{11}\cdot \omega_{11}{}^{2}+l_{IK}\cdot \sin\theta_{16}\cdot \cot\theta_{11}\cdot \omega_{16}{}^{2}+l_{IK}\cdot \cos\theta_{16}\cdot \csc\theta_{11}{}^{2}\cdot \omega_{11}\cdot \omega_{16}\\ l_{IK}\cdot \cos\theta_{16}\cdot \varepsilon_{16}+a_{3}\cdot \sin\varphi_{3}=a_{H}+l_{IK}\cdot \sin\theta_{16}\cdot \omega_{16}{}^{2} \end{array}\right.$$ (6)

**Fig 5:**
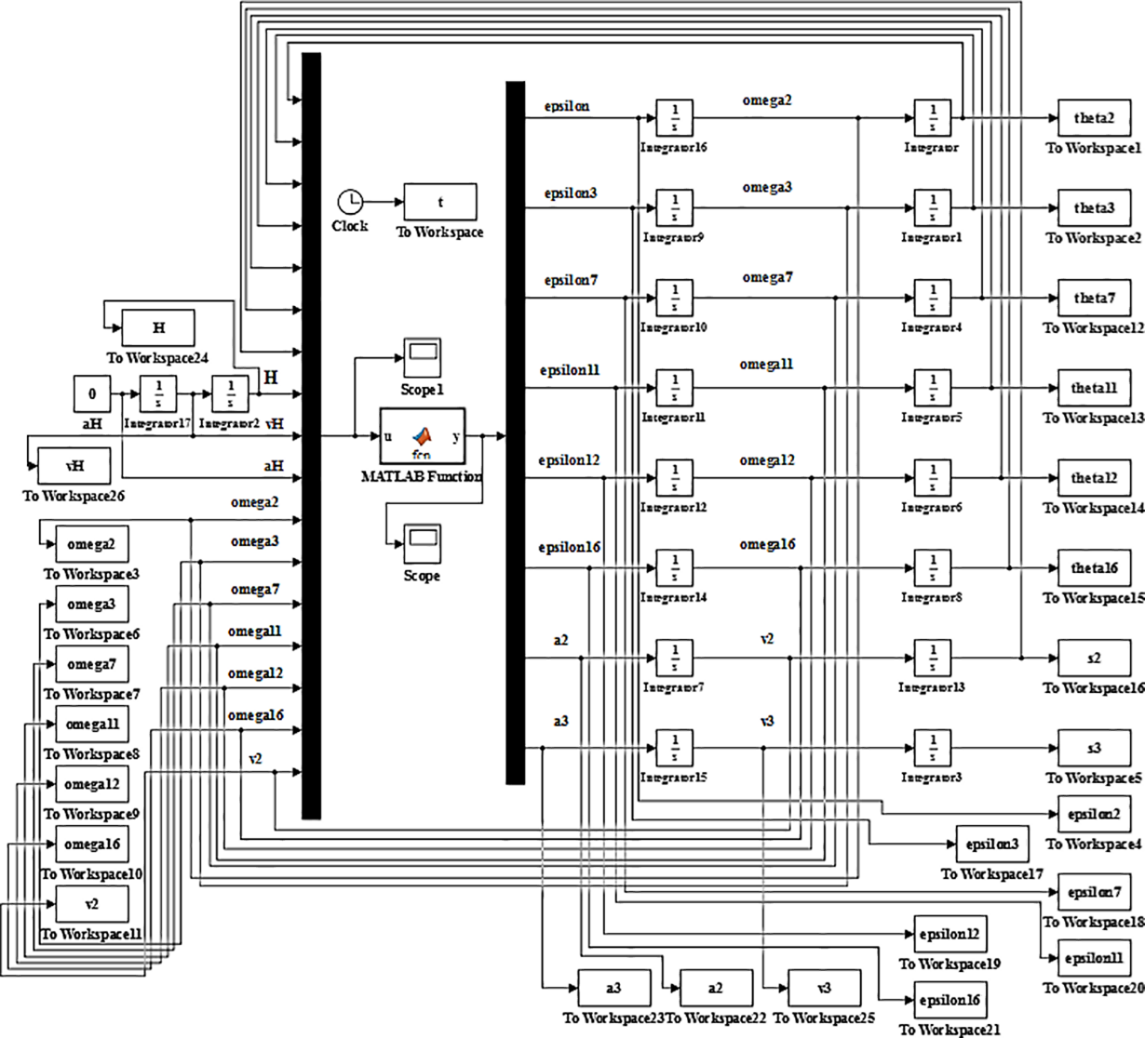
Reverse numerical solution model of the slipper-type support.

**Fig 6:**
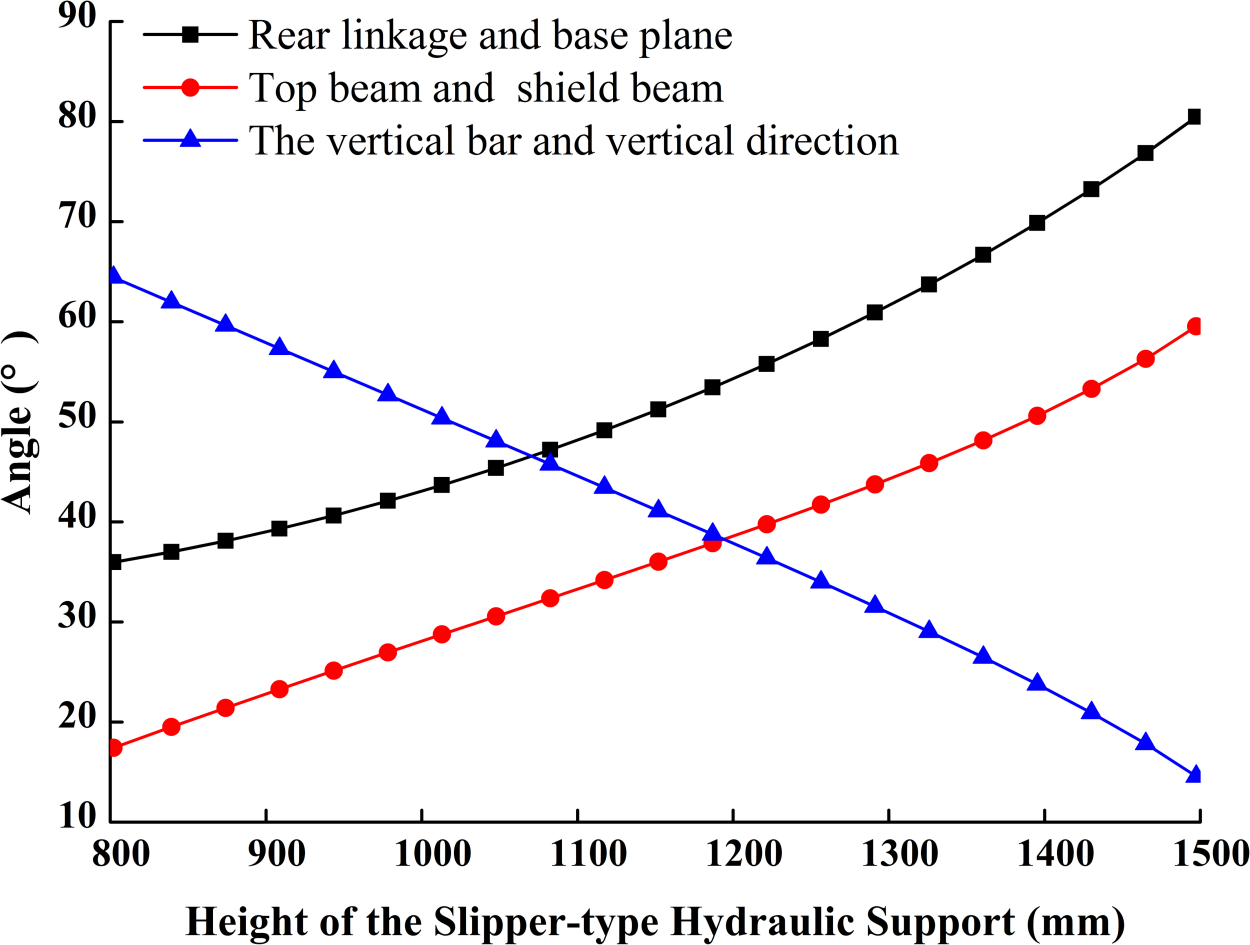
Curves of included angles between components.

**Fig 7:**
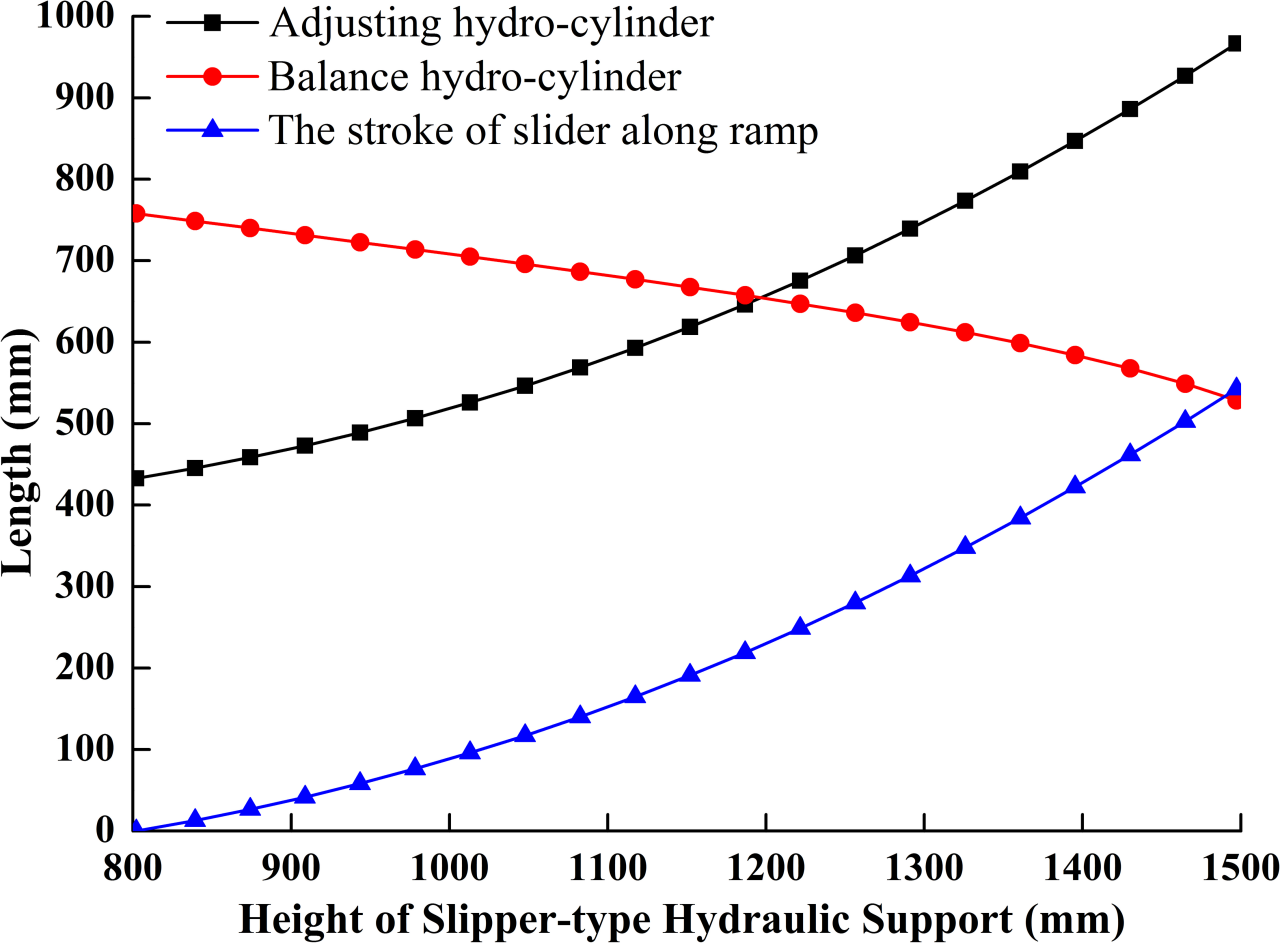
Curves of motion trail of components.

**Fig 8:**
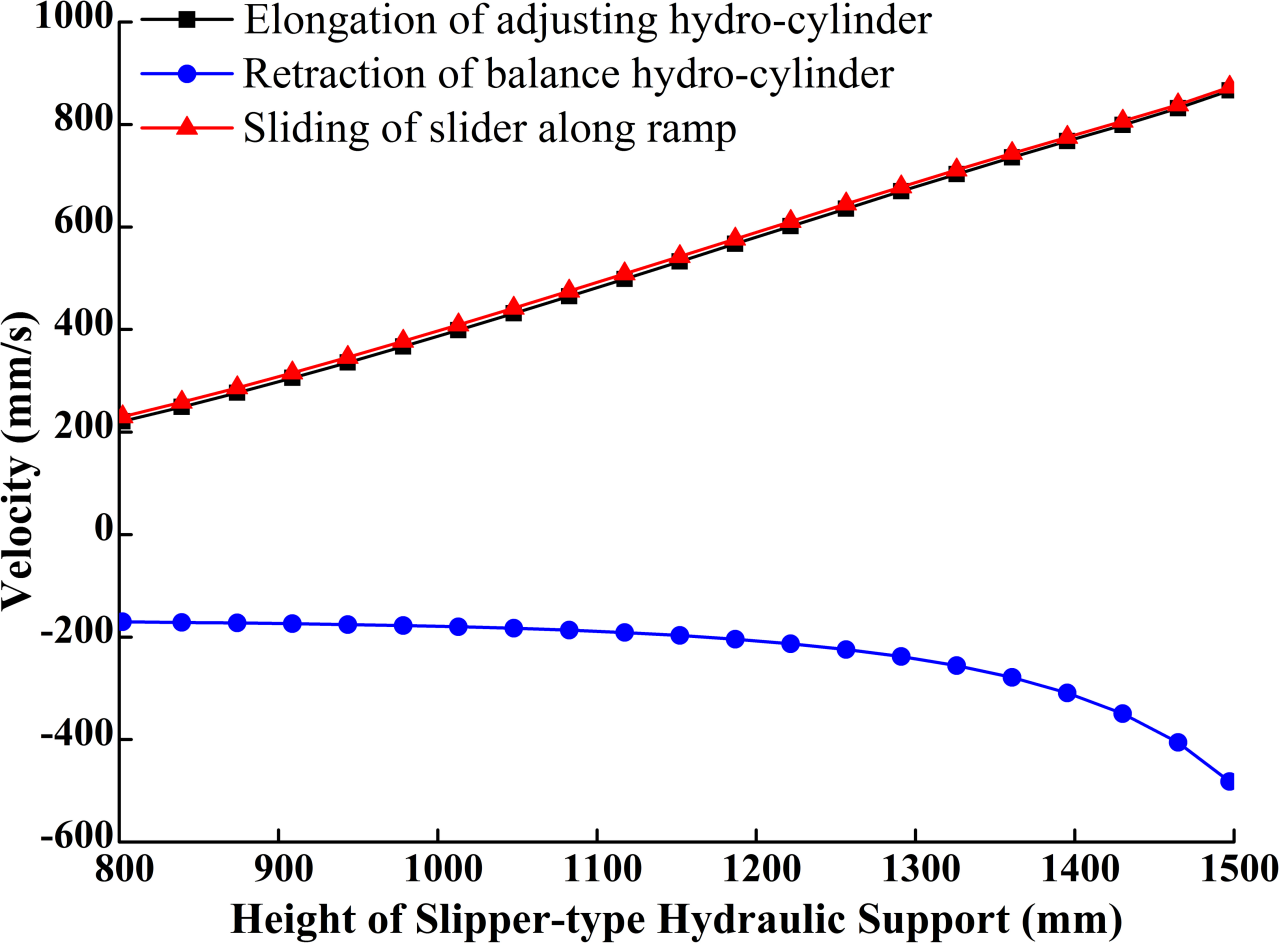
Curves of velocities of components.

**Fig 9:**
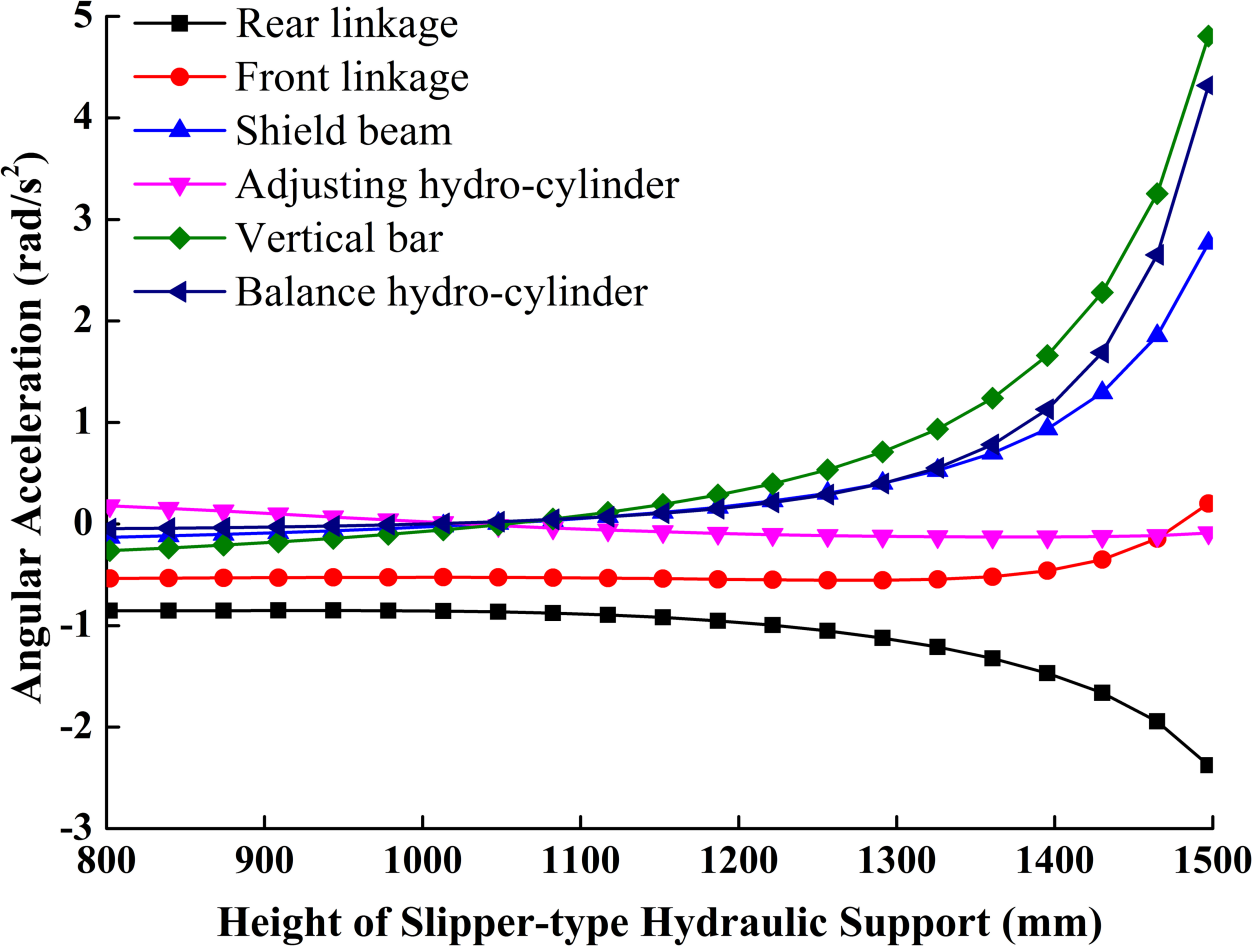
Curves of angular acceleration of components.

The simulation model (or the numerical solution model) of the slipper-type support is shown in Fig 5 with MATLAB/Simulink based on its reverse solution acceleration model. As, *ε*_2_, *ε*_3_, *ε*_7_, *ε*_11_, *ε*_12_, *ε*_16_, *a*_2_, *a*_3_ are unknown parameters and the value of *ω*_2_, *ω*_3_, *ω*_7_, *ω*_11_, *ω*_12_, *ω*_16_, *v*_2_, *v*_3_, *θ*_2_, *θ*_3_, *θ*_7_, *θ*_11_, *θ*_12_, *θ*_16_, *s*_2_, *s*_3_ obtained from integrator are set as inputs. Given driving parameters of *H*, *θ*_13_, *v*_H_, *a*_H_ and each initial amount and the rising speed *v*_H_ of the support height *H* is constant, set the angular velocities and velocities of each bar obtained from speed simulation model as the initial values of the acceleration simulation model, we obtain the changing curves of each variable in the theory model as Figs 6–9.

Fig 6 shows that the full working range of the slipper-type hydraulic support is 800~1500mm. At the lowest working position, the angle *P* between the top beam and the shield beam is about 17.5°, and the angle between the rear linkage and the base is about 36°. If the friction coefficient *W* between steel and coal gangue is 0.3, the slipper-type support meets tan*P*>*W* and *Q*≥25~30°, which can effectively prevents coal gangue from its retention at shield beam or from its falling to the back of the support. With the increase of the support height, *P* and *Q* will gradually increase and the angle between the vertical bar and vertical axis will gradually decrease. At the highest working position, the values of *P* and *Q* of the hydraulic support increase to 59.5° and 80.5° respectively, which meet the support design requirement of *P*≤52~62° and *Q*≤75~85° at the highest working position. And at this position, the dip angle will decrease to about 14°, so that the support will have both good force transmission and high supporting efficiency.

The slider added in the slipper-type support could slide along the inclined ramp on the base under the push of the adjusting hydro-cylinder. Figs 7 and 8 show that the sliding velocity of the slider is greater than the extending velocity of adjusting cylinder in a constant slight difference. With the increase of the support height, the displacement of the slider along the slipway is the same as the length changing trend of extension of adjusting hydro-cylinder, which gradually increases at an increment rate. The slider speed is very close to the elongation velocity of the adjusting hydro-cylinder. The accelerations of the slider and adjusting hydro-cylinder are comparatively stable. The two components are in an ideal stress state. When the support height increases at the constant speed *v*_H_, the balance hydro-cylinder firstly retracts at a near constant velocity. But when the support height is greater than 1300mm, the retraction velocity of the balance hydro-cylinder and the change of the velocity gradually increase. This means that the balance hydro-cylinder produces a large impact force on the whole adjusting system when the support height is greater than 1300mm.

Fig 9 shows the changing curves of angular acceleration of each component of the support in the course of the support movement from the lowest to the highest position at a constant velocity. Within the height range, the angular acceleration of the adjusting hydro-cylinder changes, but the change is close to a skew line with a small slope. This means that the adjusting cylinder receives a steady force within the height range adjusting at a constant velocity. The changes of angular acceleration of each component are slow when the support height is smaller than 1300mm. However the angular accelerations of all components, except the adjusting hydro-cylinder, increase or decrease sharply when the support height is greater than 1300mm.

The above analysis shows that when the support height is smaller than 1300mm, its components receive a steady force during height adjustment; while when the height was greater than 1300mm, its components receive relatively acute impact loads during height adjustment.

In sum, the design of the slipper-type hydraulic support is rational in terms of structure. It can be applied into the working face in the height range of 800~1500mm. The design is more suitable for coal mining working faces which are lower than 1300mm. The lifting speed of the support should be monitored when the adjusting height is greater than 1300mm.

## Load-bearing mechanical models of two different supports

Because of the irregularity of the roofs of the working face and the complicated roof contact, the load difference caused by the work environment is great. To make a quantitative analysis and comparison of the force conditions between the slipper-type and traditional supports, we make the following assumptions: (1) the top beam always contacts the roof evenly at any arbitrary height without causing unbalanced loading, torque, etc.; (2) the load varies linearly along the length of the top beam and distributes evenly along the width of the top beam, and the load F1 on the support concentrated in the 1/3 length of the top beam; (3) ignore the retractability of the hydro-cylinder due to the compressibility of hydraulic oil (i.e., assume hydraulic oil is a rigid body); (4) ignore the impact of coal gangue falling on the support; (5) ignore the weight of the support components.

### The mechanics model of traditional support

**Fig 10:**
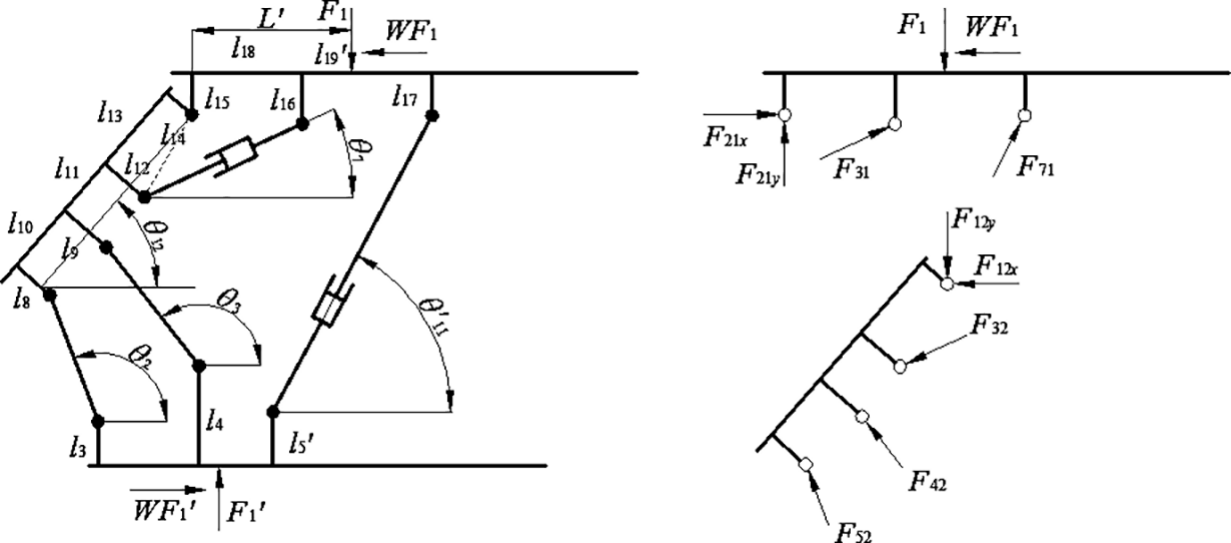
Two-dimensional model of traditional support and its mechanics model of components.

Fig 10 illustrates the mechanics model of the traditional support working at height *H*. Based on this model, we will study the load-bearing model of prop of traditional support when the top beam contacts evenly the roof. The load-bearing equilibrium equation of the traditional support at height *H* can be obtained as follows. We regard the top beam and shield beam as detached bodies as shown in Fig 10:

$$\left\{ \begin{array}{l} F_{21x}+F_{31}\cdot \cos\theta_{7}+F_{71}\cdot \cos\theta_{11}^{\prime }=F_{1}\cdot W\\ F_{21y}+F_{31}\cdot \sin\theta_{7}+F_{71}\cdot \sin\theta_{11}^{\prime }=F_{1}\\ F_{31}\cdot A+F_{71}\cdot B+F_{1}\cdot W\cdot l_{15}=F_{1}\cdot L^{\prime }\\ F_{12x}+F_{32}\cdot \cos\theta_{7}+F_{42}\cdot \cos(\pi -\theta_{3})+F_{52}\cdot \cos(\pi -\theta_{2})=0\\ F_{12y}+F_{32}\cdot \sin\theta_{7}=F_{42}\cdot \sin(\pi -\theta_{3})+F_{52}\cdot \sin(\pi -\theta_{2})\\ F_{32}\cdot C+F_{42}\cdot D+F_{52}\cdot E=0 \end{array}\right.$$ (7)

Where *F*_*ij*_ is the force on component *j* given by component *i*, *F*_*ijx*_ and *F*_*ijy*_ are decomposition of *F*_*ij*_ along *x* and *y* axis respectively, *W* is the friction coefficient between the top beam and the roof, L′ is the distance between the action spot of concentrated load *F*_1_ and the hinged joint between the shield beam and the top beam.

$$\begin{array}{l} A=\cos\theta_{7}\cdot \left(l_{16}-l_{15}\right)+\sin\theta_{7}\cdot l_{18};\;B=\cos\theta_{11}^{\prime }\cdot \left(l_{17}-l_{15}\right)+\sin\theta_{11}^{\prime }\cdot \left(l_{18}+l_{19}^{\prime }\right);\;N=-\frac{BC}{A}-\frac{D\cdot \cos\theta_{11}^{\prime }}{\cos\theta_{3}};\\ C=\cos\left(\theta_{12}-\theta_{7}\right)\cdot \left(l_{12}-l_{14}\right)+\sin\left(\theta_{12}-\theta_{7}\right)\cdot l_{13};\;D=\sin\left(\theta_{3}-\theta_{12}\right)\cdot \left(l_{11}+l_{13}\right)-\cos\left(\theta_{3}-\theta_{12}\right)\left(l_{9}-l_{14}\right);\\ E=\sin\left(\theta_{2}-\theta_{12}\right)\cdot \left(l_{10}+l_{11}+l_{13}\right)-\cos\left(\theta_{2}-\theta_{12}\right)\left(l_{8}-l_{14}\right);\;P=L^{\prime }-W\cdot l_{15};Q=\sin\theta_{2}-\tan\theta_{3}\cdot \cos\theta_{2};\\ T=\sin\theta_{11}^{\prime }-\tan\theta_{3}\cdot \cos\theta_{11}^{\prime };\;U=1-W\cdot \tan\theta_{3};\;V=-\frac{P\cdot C}{A}-\frac{D\cdot W}{\cos\theta_{3}} \end{array}$$

The serial numbers of components are: top beam-1, shield beam-2, balance jack-3, front linkage-4, rear linkage-5, base-6 and prop-7. There are the following constrains: *F*_1_′ = *F*_1_, *F*_32_ = *F*_31_, *F*_12*x*_ = *F*_21*x*_, *F*_12*y*_ = *F*_21*y*_. Thus, the above equation could be solved and obtain that:

$$F_{71}=\gamma \cdot F_{1},\gamma =\left(\frac{VQ-MU}{NQ-MT}\right)$$ (8)

$$F_{52}=\alpha \cdot F_{1},\alpha =\left[\frac{U\cdot \left(NQ-MT\right)-T\cdot \left(VQ-MU\right)}{Q\cdot \left(NQ-MT\right)}\right]$$ (9)

$$F_{42}=\beta \cdot F_{1},\beta =\left[\frac{W}{\cos\theta_{3}}-\frac{U\cdot \left(NQ-MT\right)-T\cdot \left(VQ-MU\right)}{Q\cdot \left(NQ-MT\right)}\cdot \frac{\cos\theta_{2}}{\cos\theta_{3}}-\frac{VQ-MU}{NQ-MT}\cdot \frac{\cos\theta_{11}^{\prime }}{\cos\theta_{3}}\right]$$ (10)

$$F_{31}=\chi \cdot F_{1},\chi =\left[\frac{P}{A}-\left(\frac{VQ-MU}{NQ-MT}\right)\cdot \frac{B}{A}\right]$$ (11)

$$F_{21y}=\delta \cdot F_{1},\delta =\left[1-\left(\frac{P}{A}-\frac{VQ-MU}{NQ-MT}\cdot \frac{B}{A}\right)\cdot \sin\theta_{7}-\left(\frac{VQ-MU}{NQ-MT}\right)\cdot \sin\theta_{11}^{\prime }\right]$$ (12)

$$F_{21x}=\zeta \cdot F_{1},\zeta =\left[W-\left(\frac{P}{A}-\frac{VQ-MU}{NQ-MT}\cdot \frac{B}{A}\right)\cdot \cos\theta_{7}-\left(\frac{VQ-MU}{NQ-MT}\right)\cdot \cos\theta_{11}^{\prime }\right]$$ (13)

Where the elongation of prop is *l*_*s*_, and γ, α, β, χ, δ and ζ is the scale coefficient.

The parameters, such as angles, could be calculated when the position of support is given. We take sampling with an interval of 50mm in the vertical direction within the full load-bearing range (leaving out the limit height point). We get 13 groups of angle values and set them into the Eq. (8)-(13) and obtain the data listed in Table 1.

**Table 1:** Location parameters of traditional support.

*h*/*mm*	850	900	950	1000	1050	1100	1150	1200	1250	1300	1350	1400	1450
***θ***_**2**_**/**°	142.6	140.9	138.9	136.7	134.2	131.4	128.4	125.1	121.5	117.5	113.2	108.5	103.3
***θ***_**3**_**/**°	167.2	164.5	161.7	158.8	155.7	152.4	149.0	145.4	141.6	137.7	133.5	129.3	124.9
***θ***_**7**_**/**°	8.95	10.4	11.8	13.2	14.6	16.0	17.5	18.9	20.4	22.1	23.8	25.8	28.2
**θ**_**11**_′	34.6	37.9	40.2	42.4	44.5	46.4	48.1	49.7	51.2	52.5	53.8	55.1	56.4
***θ***_**12**_**/**°	13.3	16.0	18.8	21.4	24.1	26.7	29.4	32.1	35.0	38.0	41.3	45.0	49.3
***γ***	1.77	1.63	1.53	1.45	1.39	1.33	1.29	1.25	1.22	1.20	1.18	1.17	1.17
***α***	-0.63	-0.65	-0.68	-0.70	-0.71	-0.72	-0.72	-0.728	-0.73	-0.73	-0.74	-0.73	-0.73
***β***	1.70	1.54	1.45	1.37	1.30	1.24	1.18	1.13	1.08	1.04	1.00	0.95	0.90
***χ***	-0.45	-0.38	-0.32	-0.27	-0.22	-0.18	-0.15	-0.13	-0.11	-0.10	-0.10	-0.11	-0.14
***Δ***	0.06	0.07	0.076	0.081	0.083	0.085	0.086	0.086	0.086	0.086	0.087	0.088	0.093
**ζ**	-0.708	-0.609	-0.554	-0.510	-0.474	-0.444	-0.416	-0.390	-0.365	-0.338	-0.307	-0.271	-0.225
***l***_***s***_	23.5	49.3	77.3	107.4	139.7	174.0	210.1	247.9	287.1	327.4	368.2	408.7	447.6

### The mechanics model of slipper-type support

**Fig 11:**
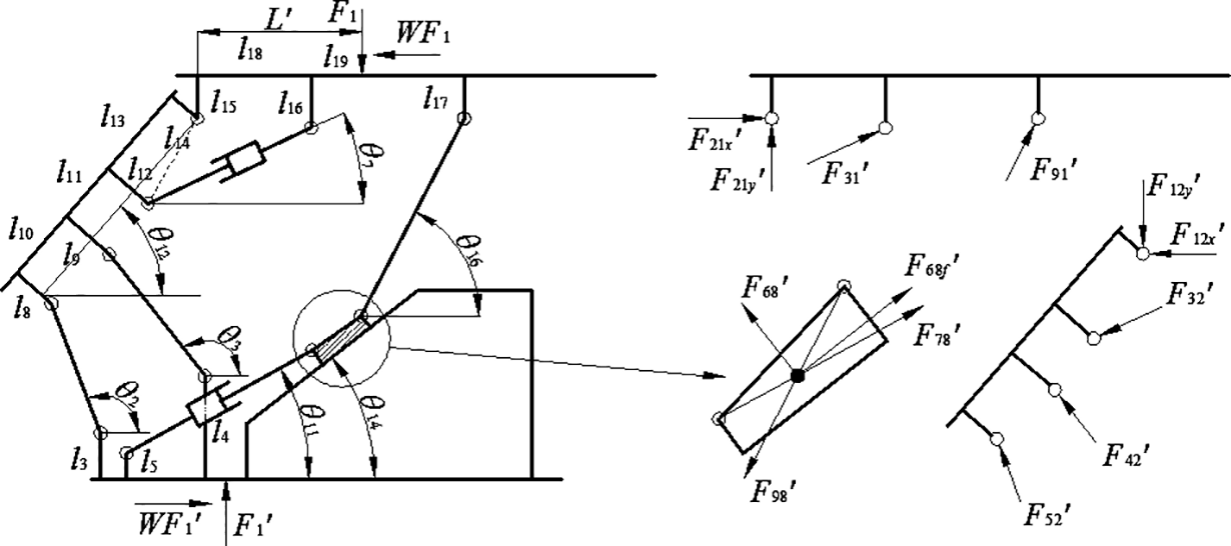
2D model of slipper-type support and its mechanical model of components.

The slipper-type support changes the prop support into joint multi-body load-transferring support composed of vertical bar, slider, adjusting hydro-cylinder and inclined ramp. Fig 11 illustrates the mechanics model of the slipper-type support.

The load-bearing equilibrium equation of the slipper-type support at height *H* could be obtained as follows. We regard the top beam and shield beam as detached bodies as well.

$$\left\{ \begin{array}{l} F_{21x}^{\prime }+F_{31}^{\prime }\cdot \cos\theta_{7}+F_{91}^{\prime }\cdot \cos\theta_{16}=F_{1}\cdot W\\ F_{21y}^{\prime }+F_{31}^{\prime }\cdot \sin\theta_{7}+F_{91}^{\prime }\cdot \sin\theta_{16}=F_{1}\\ F_{31}^{\prime }\cdot A+F_{91}^{\prime }\cdot B^{\prime }+F_{1}\cdot W\cdot l_{15}=F_{1}\cdot L^{\prime }\\ F_{12x}^{\prime }+F_{32}^{\prime }\cdot \cos\theta_{7}+F_{42}^{\prime }\cdot \cos(\pi -\theta_{3})+F_{52}^{\prime }\cdot \cos(\pi -\theta_{2})=0\\ F_{12y}^{\prime }+F_{32}^{\prime }\cdot \sin\theta_{7}=F_{42}^{\prime }\cdot \sin(\pi -\theta_{3})+F_{52}^{\prime }\cdot \sin(\pi -\theta_{2})\\ F_{32}^{\prime }\cdot C+F_{42}^{\prime }\cdot D+F_{52}^{\prime }\cdot E=0 \end{array}\right.$$ (14)

The serial numbers of components are: top beam-1, shield beam-2, balance jack-3, front linkage-4, rear linkage-5, base-6, adjusting hydro-cylinder-7, slider-8 and vertical bar-9.

There are the following constrains: *F*_1_′ = *F*_1_, *F*_21*x*_′ = *F*_12*x*_′, *F*_21y_′ = F_12y_′, *F*_31_′ = *F*_32_′, *F*_91_′ = *F*_98_′.

Solve the equation as follows,

$$F_{91}^{\prime }=\left(\frac{VQ-MU}{N^{\prime }Q-MT^{\prime }}\right)\cdot F_{1}$$ (15)

$$F_{52}^{\prime }=\alpha^{\prime }\cdot F_{1},\alpha^{\prime }=\left[\frac{U\cdot \left(N^{\prime }Q-MT^{\prime }\right)-T^{\prime }\cdot \left(VQ-MU\right)}{Q\cdot \left(N^{\prime }Q-MT^{\prime }\right)}\right]$$ (16)

$$F_{42}^{\prime }=\beta^{\prime }\cdot F_{1},\beta^{\prime }=\left[\frac{W}{\cos\theta_{3}}-\frac{U\cdot \left(N^{\prime }Q-MT^{\prime }\right)-T^{\prime }\cdot \left(VQ-MU\right)}{Q\cdot \left(N^{\prime }Q-MT^{\prime }\right)}\cdot \frac{\cos\theta_{2}}{\cos\theta_{3}}-\frac{VQ-MU}{N^{\prime }Q-MT^{\prime }}\cdot \frac{\cos\theta_{16}}{\cos\theta_{3}}\right]$$ (17)

$$F_{31}^{\prime }=\chi^{\prime }\cdot F_{1},\chi^{\prime }=\left[\frac{P}{A}-\left(\frac{VQ-MU}{N^{\prime }Q-MT^{\prime }}\right)\cdot \frac{B^{\prime }}{A}\right]$$ (18)

$$F_{21y}^{\prime }=\delta^{\prime }\cdot F_{1},\delta^{\prime }=\left[1-\left(\frac{P}{A}-\frac{VQ-MU}{N^{\prime }Q-MT^{\prime }}\cdot \frac{B^{\prime }}{A}\right)\cdot \sin\theta_{7}-\left(\frac{VQ-MU}{N^{\prime }Q-MT^{\prime }}\right)\cdot \sin\theta_{16}\right]$$ (19)

$$F_{21x}^{\prime }=\zeta^{\prime }\cdot F_{1},\zeta^{\prime }=\left[W-\left(\frac{P}{A}-\frac{VQ-MU}{N^{\prime }Q-MT^{\prime }}\cdot \frac{B^{\prime }}{A}\right)\cdot \cos\theta_{7}-\left(\frac{VQ-MU}{N^{\prime }Q-MT^{\prime }}\right)\cdot \cos\theta_{16}\right]$$ (20)

Where $B^{\prime }=\cos\theta_{16}\cdot \left(l_{17}-l_{15}\right)+\theta_{16}\cdot \left(l_{18}+l_{19}^{\prime }\right)$, $N^{\prime }=-\frac{B^{\prime }C}{A}-\frac{D\cdot \cos\theta_{16}}{\cos\theta_{3}}$, $T^{\prime }=\sin\theta_{16}-\tan\theta_{3}\cdot \cos\theta_{16}$, the elongation of the adjusting hydro-cylinder is *l*_*s*_′.

Then we regard the slider as a detached body and build its two dimensional equilibrium equation of force system as follows:

$$\left\{ \begin{array}{l} F_{98}^{\prime }\cdot \cos\theta_{16}+F_{68}^{\prime }\cdot \sin\theta_{14}=F_{68f}^{\prime }\cdot \cos\theta_{14}+F_{78}^{\prime }\cdot \cos\theta_{11}\\ F_{98}^{\prime }\cdot \sin\theta_{16}=F_{68}^{\prime }\cdot \cos\theta_{14}+F_{68f}^{\prime }\cdot \sin\theta_{14}+F_{78}^{\prime }\cdot \sin\theta_{11} \end{array}\right.$$ (21)

Where *F*_68*f*_′ = *f*·*F*_68_′, if friction coefficient between steels is *f*. Combing with Eq (15), we can obtain:

$$F_{78}^{\prime }=\gamma^{\prime }\cdot F_{1},\gamma^{\prime }=\frac{\cos(\theta_{14}-\theta_{16})+f\cdot \sin(\theta_{14}-\theta_{16})}{\cos(\theta_{14}-\theta_{11})+f\cdot \sin(\theta_{14}-\theta_{11})}\cdot \frac{VQ-MU}{N^{\prime }Q-MT^{\prime }}$$ (22)

Finally, we take sampling with an interval of 50mm in the vertical direction within the range of 850mm-1450mm. We get 13 groups of angle values and set them into the Eqs (16)–(22) and obtain the data listed in Table 2.

**Table 2:** Location parameters of slipper-type support.

*h*/*mm*	850	900	950	1000	1050	1100	1150	1200	1250	1300	1350	1400	1450
***θ***_**2**_**/**°	142.7	140.9	138.9	136.7	134.2	131.4	128.4	125.1	121.5	117.5	113.2	108.5	103.3
***θ***_**3**_**/**°	167.2	164.5	161.7	158.8	155.7	152.4	149.0	145.4	141.6	137.7	133.5	129.3	124.9
***θ***_**7**_**/**°	8.95	10.41	11.84	13.25	14.65	16.05	17.47	18.93	20.45	22.07	23.83	25.82	28.16
***θ***_**11**_**/**°	14.89	15.55	16.25	16.97	17.69	18.40	19.10	19.77	20.42	21.03	21.56	22.13	22.62
***θ***_**12**_**/**°	13.27	16.04	18.76	21.43	24.08	26.73	29.40	32.14	34.99	38.02	41.30	44.97	49.27
***θ***_**14**_**/**°	30	30	30	30	30	30	30	30	30	30	30	30	30
***θ***_**16**_**/**°	28.74	32.07	35.38	38.68	41.99	45.29	48.60	51.95	55.33	58.80	62.40	66.21	70.41
***γ***′	1.922	1.693	1.504	1.345	1.210	1.094	0.992	0.902	0.821	0.745	0.672	0.600	0.522
***α***′	-0.678	-0.683	-0.673	-0.649	-0.613	-0.565	-0.508	-0.439	-0.360	-0.267	-0.159	-0.031	0.125
***β***′	2.037	1.809	1.670	1.429	1.261	1.103	0.951	0.802	0.652	0.499	0.339	0.168	-0.019
***χ***′	-0.765	-0.662	-0.571	-0.493	-0.427	-0.373	-0.330	-0.297	-0.272	-0.252	-0.236	-0.217	-0.184
***δ***	0.161	0.171	0.179	0.185	0.188	0.190	0.192	0.193	0.193	0.194	0.198	0.195	0.193
**ζ**′	-0.692	-0.563	-0.462	-0.380	-0.309	-0.245	-0.185	-0.127	-0.069	-0.012	0.045	0.099	0.144
***l***_***s***_′	16.7	36.9	60.2	86.8	116.6	150.0	186.9	227.3	271.2	318.6	369.4	423.2	479.9

### Loaded forces comparison and analysis of two supports

**Fig 12:**
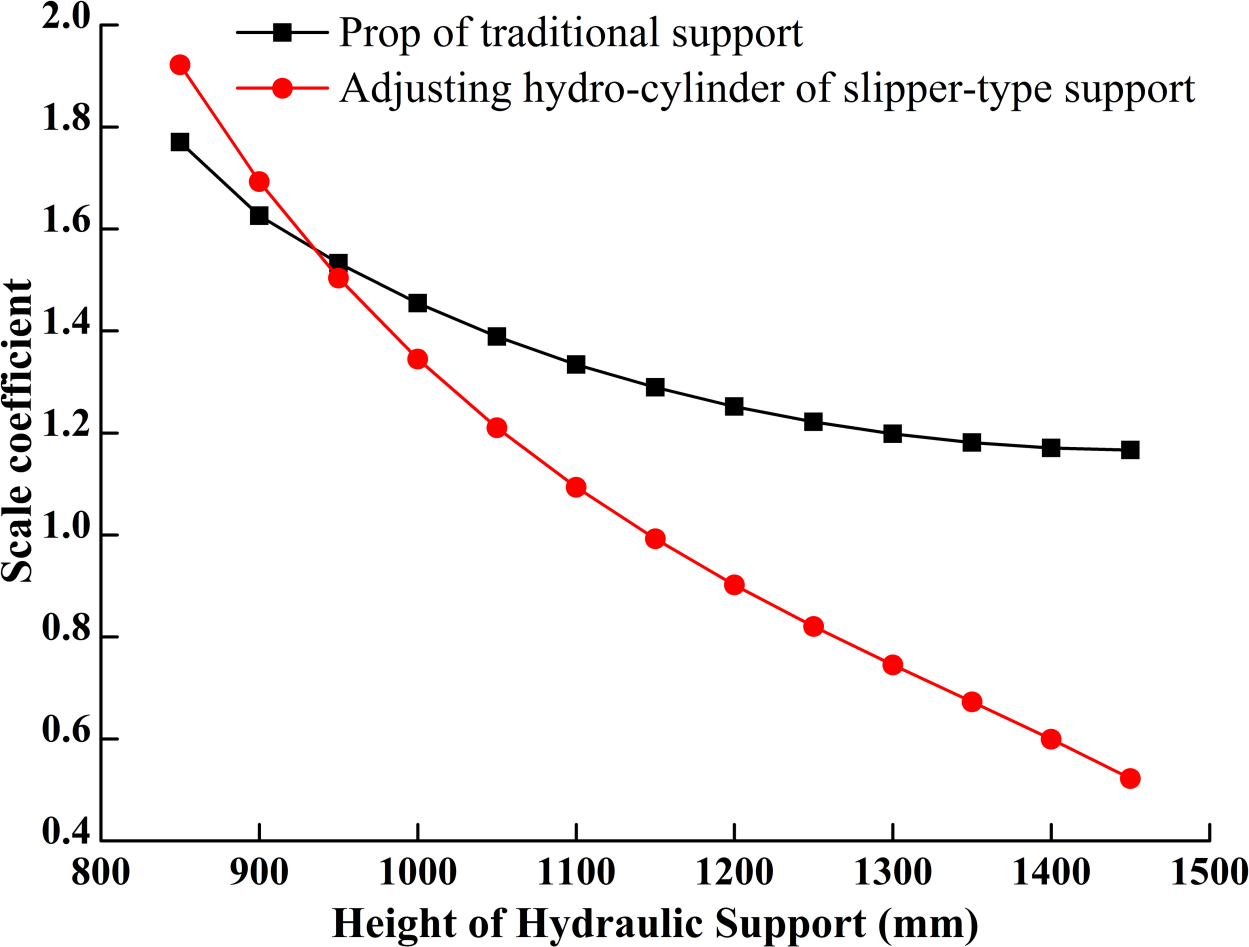
Load-bearing of driving hydro-cylinders.

**Fig 13:**
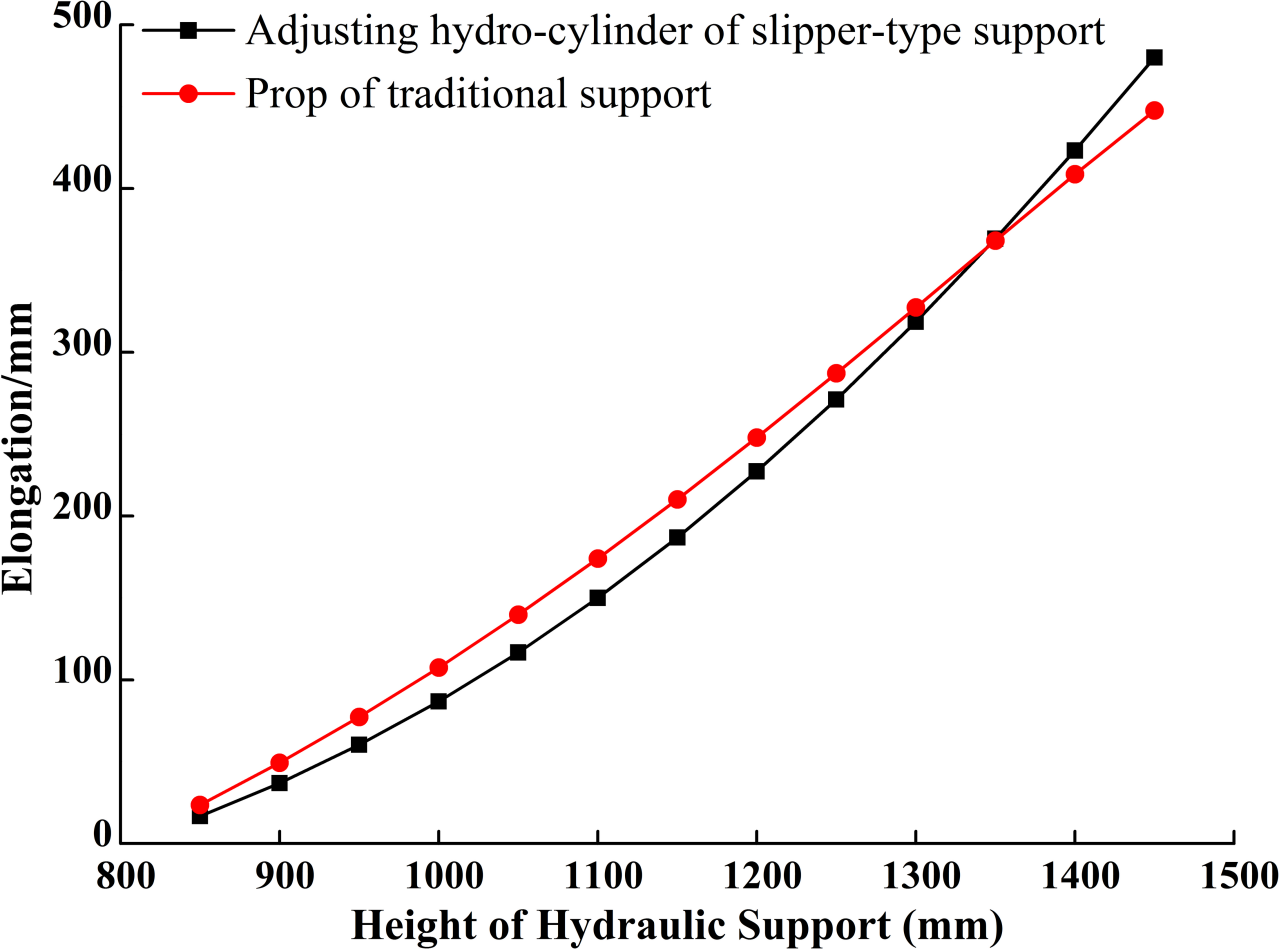
Elongation of driving hydro-cylinders.

**Fig 14:**
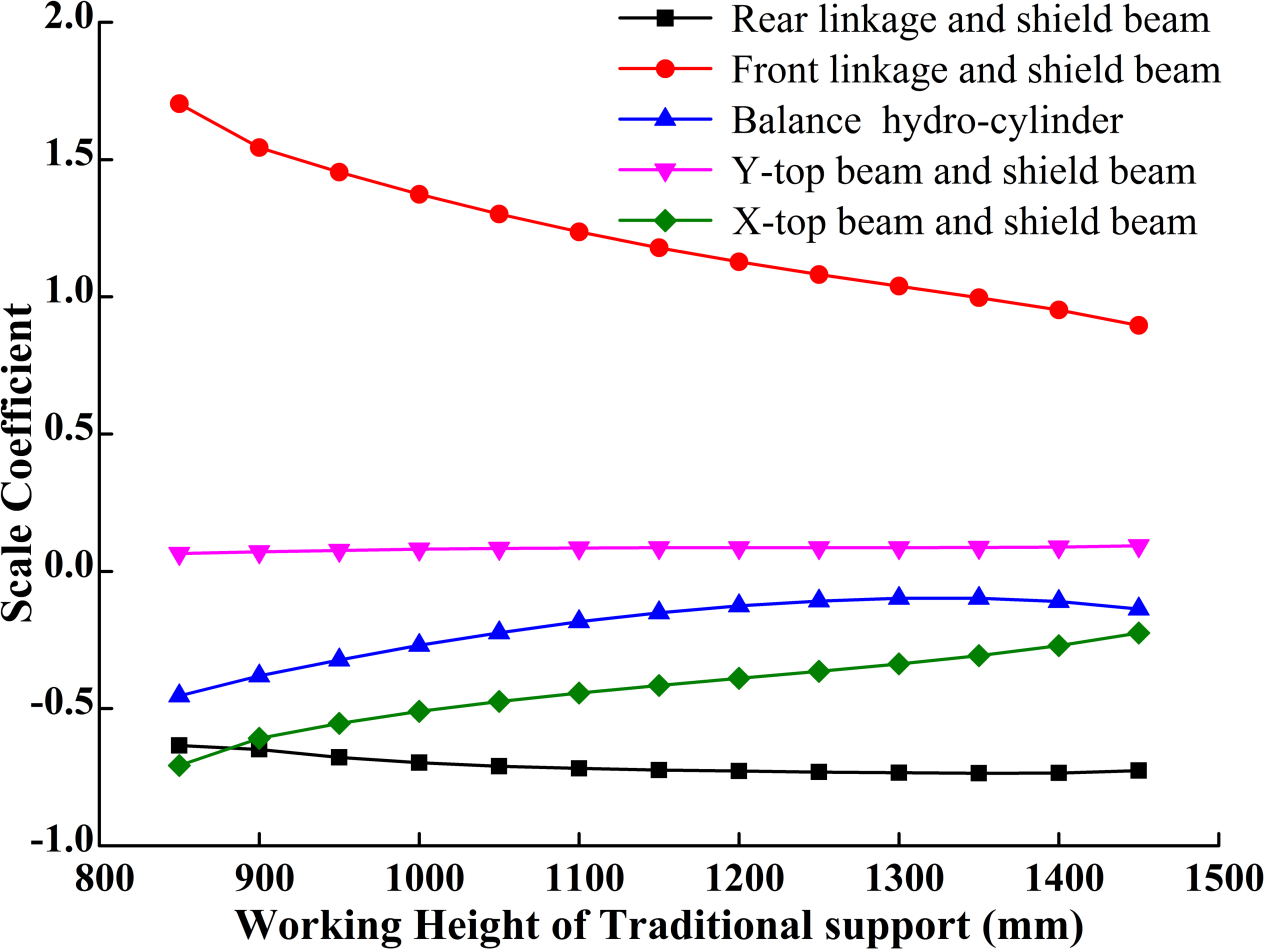
Hinge joints forces of traditional support.

**Fig 15:**
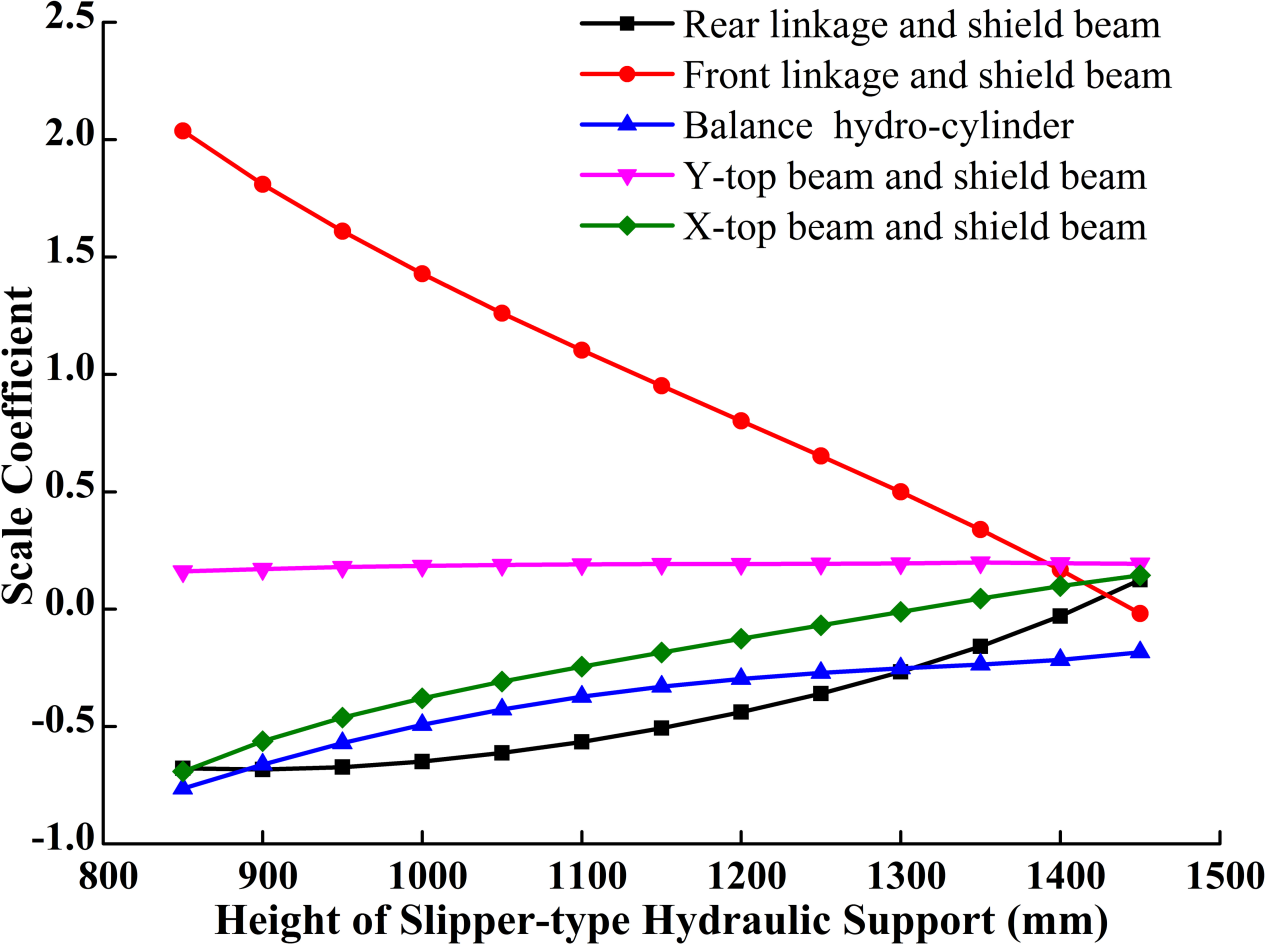
Hinge joints forces of slipper-type support.

According to the force relationships of components deduced from the load-bearing mechanic models of the slipper-type and the traditional support and the data listed in Tables 1 and 2, we have the following load-bearing curves of the two kinds of supports as shown in Figs 12–15 when the roof pressure is constantly concentrated as *F*_1_.

Fig 12 shows that with the increase of working height, the forces needed to support roofs for both supports gradually decrease and the decreasing rate gradually slows down. When the supporting height is in the range of 800~930mm, the force provided by the slipper-type support is greater than that provided by the traditional support under the same roof pressure. When the supporting height is greater than 930mm, the force provided by the slipper-type support is smaller than that provided by the traditional support under the same roof pressure. We also see that the pressure difference increases gradually as the height increases. That means: (1) the demand on oil pressure for the slipper-type support is higher than that for the traditional support when the working height is less than 930mm; (2) when the working height is greater than 930mm, the demand on oil pressure for the slipper-type support decreases gradually compared with that of traditional support when both support the same roof, and the difference in oil pressure increases gradually with the increase of the working height; (3) at the highest working position, the oil pressure demanded by the slipper-type support is only 44.7% of that demanded by the traditional support. Therefore, from the aspect of energy saving, the slipper-type support is more suitable for working faces which are in the range of 930~1500mm height. With the lower demand on oil pressure, the slipper-type support reduces the requirements for the stability and reliability of the hydraulic system and the pump station as well. If using the same hydraulic system and pump station, the slipper-type support would have higher stability and larger support force than the traditional support, and so the slipper-type support could operate under even larger roof pressure. That means our designed slipper-type support would be able to adapt to different height ranges of working face and provides better support than the traditional support.

Fig 13 shows that: (1) with the increase of working height, the extension speed of the adjusting hydro-cylinder in the slipper-type and the speed of prop in the traditional support increase gradually, and the amplitude of the speed of adjusting hydro-cylinder in the slipper-type is greater than that of the prop in the traditional support. When the working height is less than 1360mm, the elongation of the adjusting hydro-cylinder in the slipper-type is smaller than that of the prop in the traditional support. Therefore, the demand on oil supply for the slipper-type support is smaller when the working height is lower than 1360mm. Thus, the slipper-type support would save the oil supply and energy for the pump station; (2) compared to the prop in the traditional support, the elongation of the movable column of the hydro-cylinder in the slipper-type support is smaller, which could improve the load-bearing reliability and usage safety. When the working height is greater than 1360mm, the elongation of the adjusting hydro-cylinder in the slipper-type support is larger than that of the prop in the traditional support. This is caused by the small included angle between the vertical bar of slipper-type and vertical axis when working at the middle and high positions, and the small included angle could precisely improve the support efficiency. When the slipper-type support works at the middle and high positions, the demand on oil pressure is smaller, which could improve the working safety when the elongation of the movable column of the hydro-cylinder increases.

Comparing Fig 14 with Fig 15 shows that under the same roof pressure and the condition of roof contact: (1) the force on the hinge joint between the rear linkage and the shield beam in the traditional support increases gradually, but decreases in the slipper-type support; (2) the decreasing rate of the force on the hinge joint between the rear linkage and the shield beam in the slipper-type support is much larger than that in the traditional support; (2) during the overall load-bearing process, the pressure on the balance hydro-cylinder in slipper-type support is slightly larger than that in the traditional support, though the pressure on the slipper-type support is always within the failure range for the balance hydro-cylinder; and the moderate increase of the pressure on the balance hydro-cylinder is more beneficial to the load balance on the roof and energy-saving for the whole hydraulic system; (3) when the support approaches the highest working position, the forces on each hinge joint in the slipper-type support are closer to 0 than those in the traditional support, except for the balance hydro-cylinder. The decrease of the force on the hinge joints improves the reliability of the support and optimizes the force states of components in the support at high working position.

## Conclusions

This paper presents a new slipper-type support composed of vertical bar, slider, adjusting hydro-cylinder and inclined ramp. We build the reverse kinematics model for the slipper-type support with the theory of mechanisms and build the simulation model of the reverse numerical solution in MATLAB/Simulink. The results show that on the one hand the new slipper-type support meets the existing design standard and has rational motion structure, on the other hand, the new slipper-type support would produce a sharp impact load when its working height is more than 1300mm.

In order to analyze the loading features of the new slipper-type support, we build the mechanics models for the traditional support and the slipper-type support under the same load conditions, and then we deduce the mechanics relations of components in the two supports and obtain the curves of forces and motion displacement of components. According to the resulted curves and relations, when the working height is higher than 930mm, the force demanded by the adjusting hydro-cylinder in the new slipper-type support is smaller than the force demanded by the prop of the traditional support; At middle and high working positions, the new slipper-type support is not only more energy-efficient than the traditional support, but also has lower demands on oil pressure than the traditional support.
